# Optical Coherence Tomography Identifies Lower Labial Salivary Gland Surface Density in Cystic Fibrosis

**DOI:** 10.1371/journal.pone.0117517

**Published:** 2015-01-26

**Authors:** Jan K. Nowak, Ireneusz Grulkowski, Karol Karnowski, Maciej Wojtkowski, Jaroslaw Walkowiak

**Affiliations:** 1 Department of Pediatric Gastroenterology and Metabolic Diseases, Poznan University of Medical Sciences, ul. Szpitalna 27/33, 60–572 Poznan, Poland; 2 Institute of Physics, Faculty of Physics, Astronomy and Informatics, Nicolaus Copernicus University, ul. Grudziadzka 5, 87–100 Torun, Poland; The Hospital for Sick Children and The University of Toronto, CANADA

## Abstract

The labial minor salivary glands (LSGs) are easily accessible mucus-secreting structures of the alimentary tract that may provide new information on the basis of gastrointestinal complications of cystic fibrosis (CF). It was shown that they are destructed in the course of cystic fibrosis. We employed wide-field, micrometer resolution *in vivo* optical coherence tomography to assess the surface density of LSGs in 18 patients with CF and 18 healthy subjects. The median LSGs’ surface densities in CF patients, and in the control group were 4.32 glands/cm^2^ and 6.58 glands/cm^2^, respectively (p = 0.006; Mann-Whitney U test). A lower LSG surface density is a previously unrecognized CF-related pathology of the alimentary tract.

## Introduction

Cystic fibrosis (CF) is a genetic disease resulting from dysfunction of the CF transmembrane conductance regulator (CFTR) [1]. CFTR-related disruption of transmembrane ion transport affects the exocrine glands and results in intestinal, pancreatic, and hepatobiliary complications [2]. Among the mucus-secreting structures of the alimentary tract affected by CF are the labial salivary glands (LSGs) [3–6].

Optical coherence tomography (OCT) enables non-invasive visualization of the internal structures of tissues. OCT is analogous to ultrasound imaging except that it uses infrared light instead of acoustic waves [7]. High-speed OCT allows for cross-sectional and volumetric imaging of tissues at micrometer resolution *in vivo* and in real time. OCT was initially used in ophthalmology and has also found valuable applications in other medical specialties, including gastroenterology [8–9] and more recently pulmonology [10–11]. Different segments of the alimentary tract can be imaged with OCT revealing microstructural details of their mucous membranes [12].

The aim of the study was to assess the differences in LSGs’ surface density between CF patients and healthy subjects (HS) by applying a new generation of OCT technology, called swept source OCT (SS-OCT), in the *in vivo* imaging of the mucosa of the lower lip.

## Materials and Methods

### Study participants

The sample size of each group (n = 18) was determined at the planning stage assuming a parameter value difference of 30%, a standard deviation of 30%, an alpha error level of 0.05, and the power of 0.84. The inclusion criterion for patients was a diagnosis of CF made according to the Cystic Fibrosis Foundation consensus guidelines [13]. The inclusion criterion for volunteers was no acute or chronic disease. The exclusion criteria were: smoking, severe dental disease, treatment with levothyroxine, and pregnancy.

The information on genotypes and median forced expiratory volume in 1 second were collected from the Polish National Cystic Fibrosis Registry. Exocrine pancreatic insufficiency was diagnosed using fecal elastase-1 concentration measurements and quantitative stool fat assessment in order to characterize the patient population [14]. Fecal elastase-1 concentrations were assessed using ELISA (ScheBo Biotech AG, Gießen, Germany) [15]. Seventy-two-hour quantitative stool fat test was carried out using the classic Van de Kamer method [16]. The study was approved by the institutional review boards at the Nicolaus Copernicus University in Torun (KB189/2013) and at the Poznan University of Medical Sciences (414/13). The research adhered to the tenets of the Declaration of Helsinki. The nature of the study was explained in detail to all its participants prior to obtaining their written consent.

### Prototype Swept Source OCT instrument

**Fig 1:**
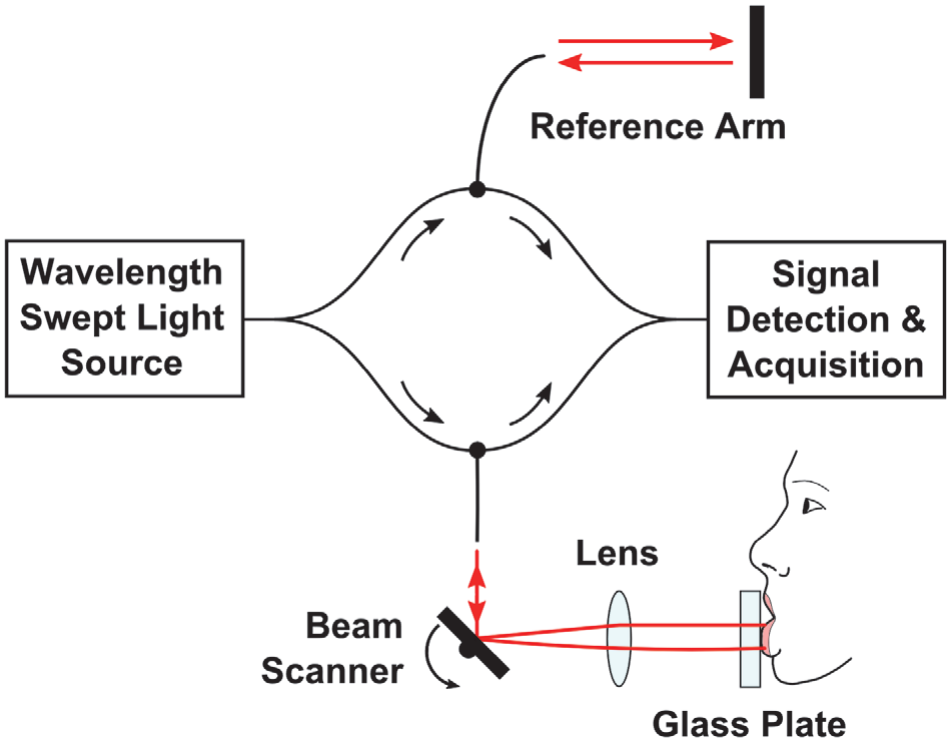
A schematic of the fiber-optic SS-OCT instrument for lip mucosa imaging.

The prototype high-speed SS-OCT instrument with a wavelength tunable light source used in the study is shown schematically in Fig. 1. The technical details of the design are given elsewhere [17]. The axial and transverse resolutions of the instrument were 6.7 μm and 20 μm in tissue, respectively. The tissue imaging depth range was 3.4 mm. The instrument had a fiber-optic interferometer configuration. The light was emitted by a wavelength swept light source engine (Axsun Technologies Inc., Billerica, MA) operating at the speed of 50,000 sweeps per second. Therefore, the SS-OCT system could acquire 50,000 A-scans (depth scans) per second. The central wavelength of the light was 1310 nm, which enabled deep penetration of light into tissue. The wavelength tuning range of ~100 nm determined high axial imaging resolution. The incident power on the mucosa was 6.2 mW at 1310 nm wavelength, which is consistent with the American National Standard Institute standards for safe exposure [18]. Two-axis scanning system in the sample arm enabled lateral beam scanning of the tissue. The sterile glass plate (individual for each participant) was included in the interface of the system to increase patient comfort and to assure uniform scanning conditions. The patient interface of the system was equipped with a sterile glass plate to enable easy exposition of the lower lip mucosa. Application of the glass plate allowed for optimization of image quality since the light focus position could be controlled. The light coming back from the reference and sample arms generated an interferometric signal. The detection and acquisition unit consisted of a dual-balance photodetector (PDB 110C; Thorlabs Inc., Newton, NJ) and a high-speed analog-to-digital card (Gage Compuscope 14200; Lachine, QC).

Interference fringes recorded in each sweep period carried information on the internal (depth) microstructure of the imaged tissue (A-scan). Therefore, transverse scanning allowed for acquisition of cross-sectional images (B-scans) and volumetric data sets. Volumetric imaging is the most comprehensive since it enables three-dimensional reconstruction of the tissue morphology. Analogous to ultrasound, OCT maps the intensity of light backscattered from tissue structures that exhibit different optical properties.

### Data acquisition and processing

During actual measurement, the subjects gently pressed the inner surface of the lower lip against a sterile glass plate. Three wide-field volumetric OCT data sets consisting of 400×400 A-scans were obtained in each participant by raster scanning over a 1.73 × 1.73 cm^2^ area of the lower lip, yielding a useful imaging surface of 2.43 cm^2^, which was limited by the lens aperture. The participants removed the head from the chinrest between imaging sessions. Instruction containing the same set of information was provided to all volunteers before their imaging sessions. The comfort level was self-assessed by the participants immediately after OCT scanning using a 5-item Likert scale (1 = very bad; 5 = very good).

**Fig 2:**
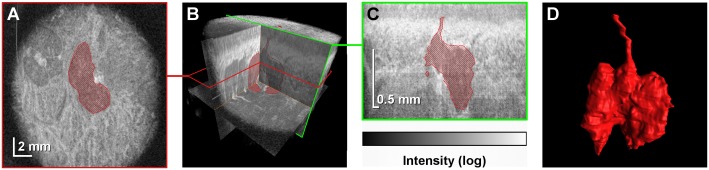
Identification of a single LSG in a volumetric data set obtained using OCT. (*A*) A projection image (C-scan) extracted from the volumetric data set. The LSG is indicated in red. (*B*) Virtually any section can by extracted from a volumetric OCT data set. (*C*) A cross-sectional image (B-scan) extracted from the volumetric data set illustrates the high axial resolution of the method, which allows for excretory duct recognition. (*D*) A three-dimensional reconstruction of the microstructure of the segmented LSG along with its excretory duct.

It took 3.2 seconds to acquire a single volumetric data set that consisted of 400×400 A-scans (Fig. 2). Each data set was post-processed to generate a set of B-scans. To generate projection images (C-scans), the mucosal surface was segmented and a tissue flattening procedure was implemented. Three-dimensional (3-D) rendering of the glands was performed using a 3-D data visualization platform (Amira 5.0; FEI Visualization Sciences Group, Burlington, MA) (S1 Video).

### LSG identification and statistical analysis

LSGs were manually identified in OCT images using Fiji image processing software (Fig. 2) [19]. An interactive pen display was used to navigate the volumetric datasets and to delineate the borders of the individual LSG for 3-D gland reconstruction. In order to perform quantitative analysis, OCT B-scans were analyzed for the presence of the excretory ducts and the glandular tissue. OCT C-scans were then used to verify the initial findings and the LSGs were counted thereafter. In cases of foci of glandular tissue clearly visible at the borders of the useful imaging surface the presence of an excretory duct was not required for LSG identification. Apart from this exception foci of glandular tissue visible on the C-scans could be matched to individual excretory ducts. Intraobserver and interobserver reproducibilities of the gland identification method assessed by calculating the intraclass correlation coefficient were 0.89 and 0.80, respectively. A statistical analysis was carried out using STATISTICA 10 (StatSoft Inc., Tulsa, OK). The Mann—Whitney U test was applied to compare distributions of parameters between the studies groups.

## Results

Thirty-six volunteers were enrolled for the study, including 18 patients with CF (Table 1). The diagnosis of CF was made on the basis of clinical presentation, sweat test results, and genetic testing according to the aforementioned guidelines [13]. The patients had the following genotypes: F508del/F508del (c.1521_1523delCTT/c.1521_1523delCTT; n = 10), F508del/3849+10kB C>T (c.1521_1523delCTT/c.3717+12191C>T; n = 4), F508del/unknown (c.1521_1523delCTT/unknown; n = 2), F508del/CFTRdel2,3 (c.1521_1523delCTT/c.54–5940_273+10250del21kb; n = 1), CFTRdel2,3/3849+10kB C>T (c.54–5940_273+10250del21kb/c.3717+12191C>T; n = 1). Patients’ median forced expiratory volume in 1 second was 54% [interquartile range: 36–71%]. Fifteen patients (83%) were diagnosed with exocrine pancreatic insufficiency. On a scale from 1 to 5 (1 being the worst, 5 being the highest), the median [interquartile range] rating of participant comfort level during the examination was 5.0 [5.0–5.0].

**Table 1:** Group characteristics. Median values and interquartile ranges are presented.

Parameter	CF patients	HS
Number	18	18
Female:male ratio	14:4	14:4
Age, years	23.8 [21.0–30.7]	26.8 [24.5–27.3]
Body mass, kg	56.3 [50.4–59.3]	57.0 [53.3–69.3]
Height, kg	165.5 [162.3–171.8]	171.0 [165.0–173.0]

CF—cystic fibrosis, HS—healthy subjects

**Fig 3:**
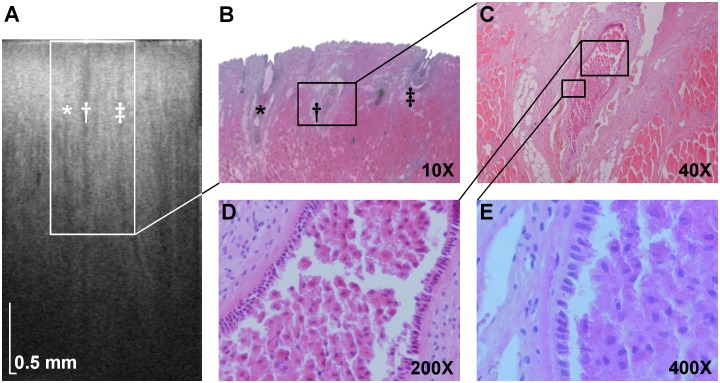
Cross-sectional OCT image of the mucosa of a porcine lower lip and corresponding histology images. (*A*) Three labial minor salivary gland excretory ducts are indicated in the OCT image (*, †, ‡). (*B*) The excretory ducts are visible in the histology sample stained with hematoxylin and eosin (10X magnification). Images obtained with magnifications of 40X (*C*), 200X (*D*), and 400X (*E*) reveal typical morphological characteristics of labial minor salivary gland excretory ducts, including the simple columnar epithelium. The ductal lumen is filled with cellular debris.

**Fig 4:**
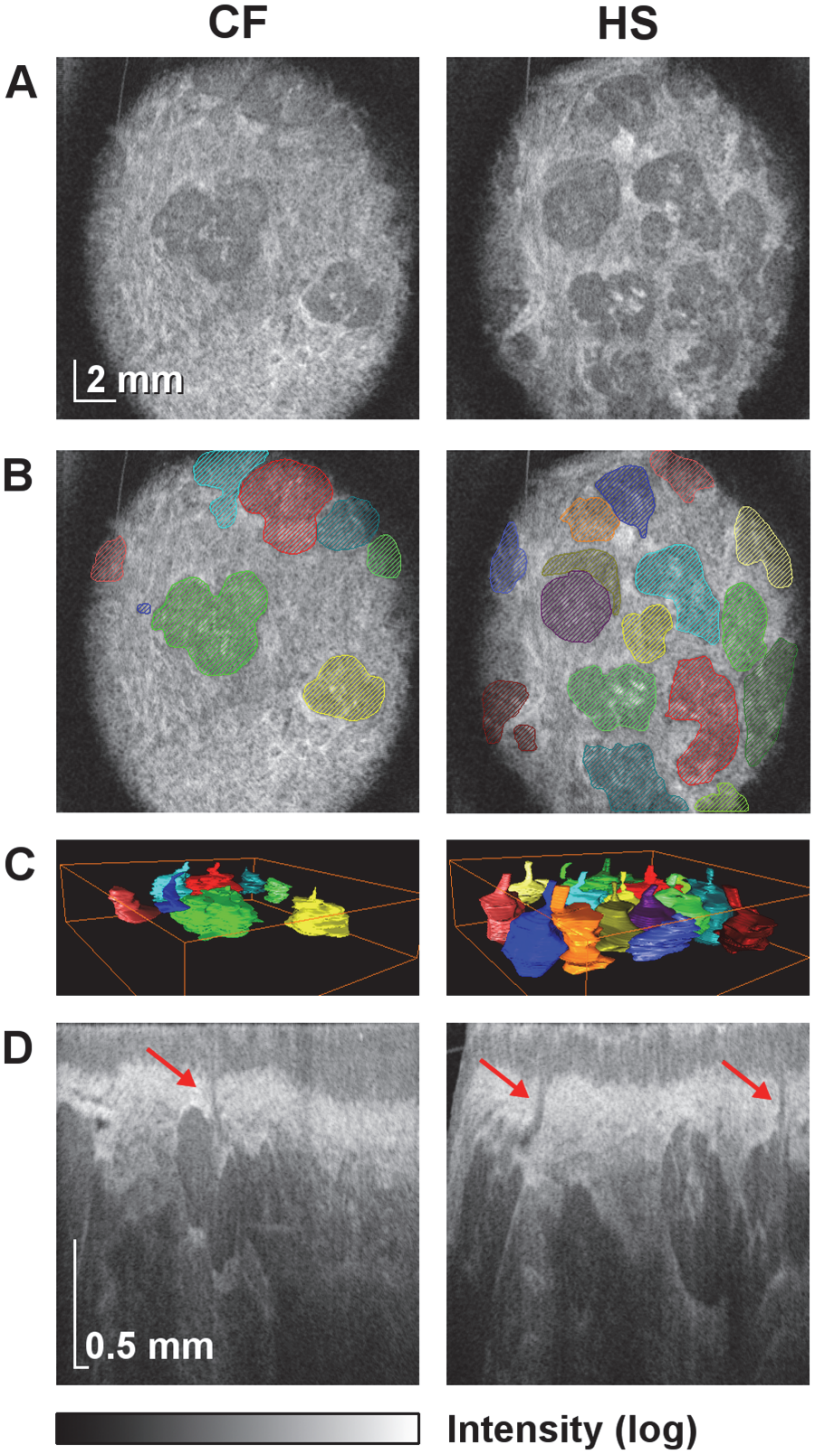
Wide-field OCT imaging of LSGs in a patient with CF (No. 11) and in a HS (No. 30). LSGs’ surface densities (4.11 and 6.58 glands/cm^2^) are representative of their respective groups. (*A*) Projection images (C-scans) of the mucosa of the central part of the lower lip at the depth of 750 μm. The intensity of signal from the glandular tissue is lower compared to the surrounding muscular tissue. (*B*) The C-scans with superimposed areas indicating individual LSGs. (*C*) Three-dimensional rendering of the segmented LSGs illustrates the difference in LSGs’ surface density between the subjects. (*D*) Sample cross-sectional images (B-scans). High axial resolution allows for identification of the LSGs and their excretory ducts (indicated by arrows).

Volumetric SS-OCT enabled non-invasive wide-field”optical biopsy” of LSGs demonstrating their comprehensive microarchitectural details. Previous studies have shown that OCT can detect glands in different parts of the alimentary tract as hypointense area, which were later identified as glandular structures by conventional histology technique [20–22]. To confirm accurate detection of LSG excretory ducts, OCT images were compared to histological sections of porcine lower lip (Fig. 3). The structures initially identified as LSG excretory ducts by OCT were confirmed as such by histology, validating the gland identification method used in this study. Images of patient lower lip region revealed a layered structure of the mucosa in the cross-sectional images (B-scans), where epithelium and connective tissue could be distinguished. The glandular tissue appeared as hypointensive compared to the surrounding highly reflective muscular tissue. Such appearance of the glandular tissue in OCT is consistent with high water content in the glandular cells [23]. Three-dimensional reconstructions, projection images, and cross-sectional images of LSGs in a patient with CF and in a HS are shown in Fig. 4. These examples are representative of their respective groups. Excretory ducts and the glandular tissue are clearly discernible in OCT images due to differences in optical properties, allowing for identification of individual LSGs.

**Fig 5:**
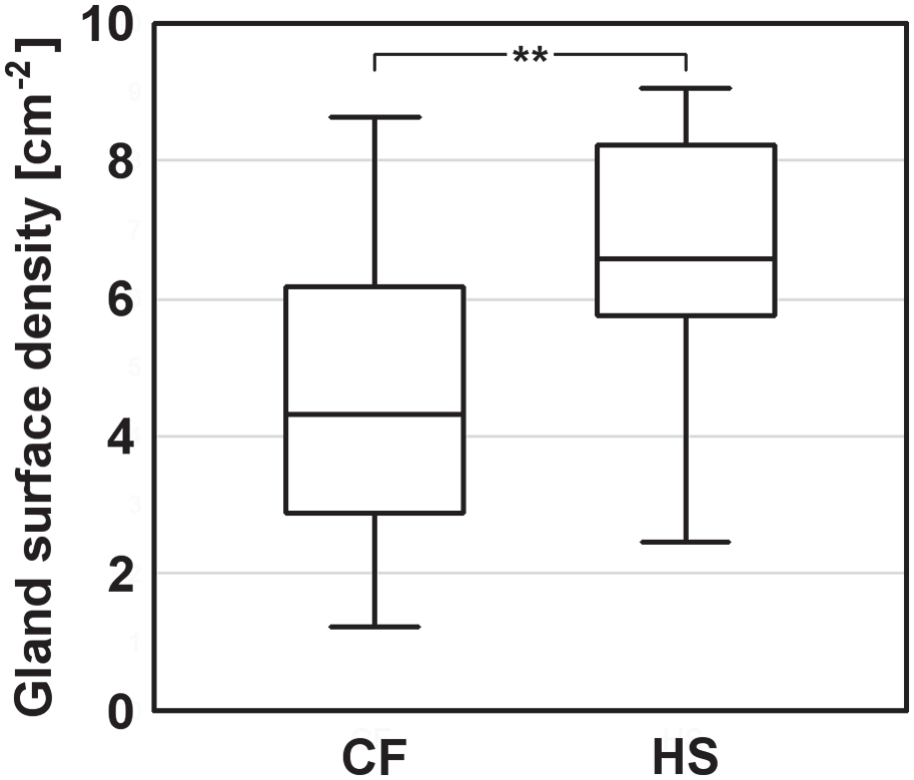
Box plot of LSGs’ surface density in CF patients and HS. Median values, interquartile ranges, and the minimum and maximum values are indicated. The asterisks denote statistical significance (P = .006).

Based on quantitative and statistical analysis of OCT data (the dataset is publicly available [24]) it has been found that LSGs’ surface density in CF patients was lower than in HS. The median [interquartile range] values were 4.32 glands/cm^2^ [2.88–6.17] and 6.58 glands/cm^2^ [5.76–8.23], respectively. The comparison of LSGs’ surface density in CF patients and HS revealed an effect of group (P = .006) describing the observed differences (Fig. 5).

## Discussion

The pathophysiological basis for the above finding is only partially explained by the available evidence. Either a lower number of LSGs develop in patients with CF in the first place, or they are lost in course of the disease. A decreased radioreceptor assay reactivity of epidermal growth factor in the saliva of CF patients argues in favor of the former hypothesis [25]. However, CFTR-related LSG dysfunction [26], [27] and the alterations in its ultrastructure revealed by electron microscopy studies [28] lend support to the latter explanation. Whether other excretory glands of the alimentary tract in patients with CF are affected in a similar manner remains to be determined. Intraluminal OCT would present many advantages in approaching this problem. Further research in the area may yield important information on causes of gastrointestinal CF complications.

In conclusion, wide-field *in vivo* OCT imaging of the mucosa of the lower lip enabled identification of a lower LSG surface density in patients with CF, which is a previously unrecognized CF-related pathology of the alimentary tract.

## Supporting Information

S1 Video OCT allows for three-dimensional reconstruction of the morphological features of the LSGs. (MP4)
